# Supplementary material for: A Humanized Yeast Phenomic Model of Deoxycytidine Kinase to Predict Genetic Buffering of Nucleoside Analog Cytotoxicity
Source: Genes (Basel). 2019 Sep 30;10(10):770. doi: 10.3390/genes10100770 (PMC6826991; doi:10.3390/genes10100770)
Supplement: Supplementary file 1 [file genes-10-00770-s001.zip › Additional_Files/Additional_File7_GOTermHeatmaps.zip!/Additional_File7_GOTermHeatmaps/meiotic_DNA_double-strand_break_processing.pdf]

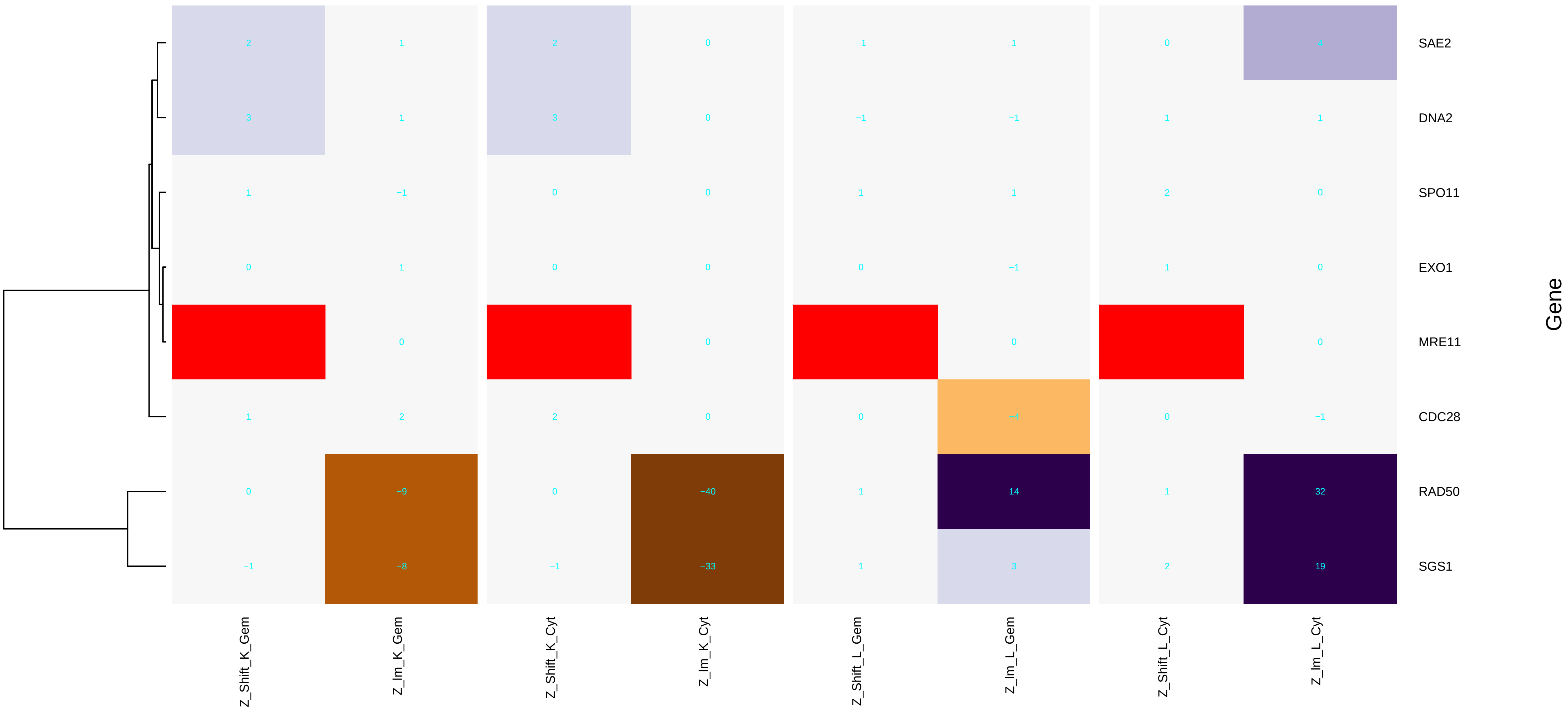
SAE2
DNA2
SPO11
EXO1
MRE11
CDC28
RAD50
SGS1
Gene
Z_Shift_K_Gem
Z_Im_K_Gem
Z_Shift_K_Cyt
Z_Im_K_Cyt
Z_Shift_L_Gem
Z_Im_L_Gem
Z_Shift_L_Cyt
Z_Im_L_Cyt
2 1 2 0 −1 1 0 4
3 1 3 0 −1 −1 1 1
1 −1 0 0 1 1 2 0
0 1 0 0 0 −1 1 0
0 0 0 0
1 2 2 0 0 −4 0 −1
0 −9 0 −40 1 14 1 32
−1 −8 −1 −33 1 3 2 19
